# Improving diet quality for female workers through market innovations: evidence from Assam’s tea estates

**DOI:** 10.3389/fnut.2025.1608153

**Published:** 2025-09-18

**Authors:** Shantanu Das, Akash Porwal, Anshita Sharma, Fred Wangila, Eric Djimeu Wouabe, Carrel Fokou, Aishwarya Choubey, Kris Woltering, Mduduzi Mmbuya, Christina Nyhus Dhillon

**Affiliations:** ^1^IPE Global, New Delhi, India; ^2^Global Alliance for Improved Nutrition, Nairobi, Kenya; ^3^Independent Consultant, Washington, DC, United States; ^4^Independent Consultant, Yaoundé, Cameroon; ^5^Global Alliance for Improved Nutrition, New Delhi, India; ^6^Global Alliance for Improved Nutrition, Utrecht, Netherlands; ^7^Global Alliance for Improved Nutrition, London, United Kingdom; ^8^Global Alliance for Improved Nutrition (GAIN), Geneva, Switzerland

**Keywords:** malnutrition, healthy diet, workforce nutrition, vegetable, dietary diversity

## Abstract

**Introduction:**

Despite the economic significance of the tea industry in India, its female workers suffer from high rates of malnutrition, particularly anemia. A three-year intervention aimed to improve diets among tea workers through behavior change and supply-side strategies across tea estates in Assam, India. Key activities included community-based edutainment, cooking demonstrations, and a market-based approach to increase access to nutritious foods via door-to-door entrepreneurs or local shops.

**Methods:**

The study used a mixed method, repeated cross-sectional design. Baseline (Feb–Mar 2021) and endline (Mar–Apr 2023) data were collected via household surveys (989 tea workers, 66 clusters), 20 key informant interviews, and 10 focus group discussions. A multi-stage sampling design selected 30 estates, 66 divisions, and 15 households per division. Women aged 15–49 were the primary respondents. Monitoring data tracked coverage and outputs. Propensity score matching controlled for differences in selected households.

**Results:**

The estimated intervention effect was a 38.3 percent increase in the consumption of vitamin A-rich fruits and vegetables and a 13.2 percentage point increase in the consumption of fortified cooking oil. Overall, this resulted in a 28.2 percentage point increase in the proportion of women meeting a minimally diverse diet. Some differences were seen across the market-based models.

**Conclusion:**

The intervention’s combination of supply-side innovations and behavior change efforts was effective and highlighted the potential for market-based approach to positively transform food environments in low-income settings. Future research should explore the long-term sustainability of such market-based interventions in improving nutritious foods consumption.

## 1 Introduction

The Indian tea industry is the country’s largest private employer, with a significant concentration of workers in Assam, Kerala, Tamil Nadu, and West Bengal. Assam, a state in the northeast, produces half of India’s tea and employs over a million workers, the majority of whom are women 26–45 years of age (1–3). Community-based studies have found very high prevalences of anemia among women in tea estates in Assam, ranging from 88 to 100% (4). An “estate” in this context refers to a self-contained, large-scale tea plantation that includes the cultivated land, a processing factory, and housing and basic amenities for its tea workers. Even among Assam’s general female population 66% are affected by iron deficiency and from 2015 and 2020 anemia rates doubled from 36 to 68% (5). Even men suffer relatively high rates of anemia (36.6%). Among women, 18% are underweight and 15% are obese while 35% of children under 5 have chronic malnutrition—also known as stunting (6, 7).

Inadequate dietary practices explain much malnutrition. Only 8% of children 6–23 months of age in Assam receive minimally adequate diets, and breastfeeding rates are very low at only 8.7% (6). The effects of poor diets have widespread repercussions leading to various health outcomes and socio-economic disparities (8). Chronic malnutrition not only compromises physical well-being but also hinders cognitive development and educational achievement, perpetuating intergenerational cycles of poverty (9). In addition, malnourished tea workers are more vulnerable to occupational hazards and infectious diseases, further undermining their ability to thrive within demanding work environments. The nutritional status of workers is closely linked with economic outcomes like productivity and absenteeism (10, 11). Among the tea workers in Assam limited nutrition awareness, inadequate resources, and systemic inequalities hamper the consumption of healthy diets. Reliance on high-calorie but low-nutrient foods coupled with the high cost of fresh produce results in unbalanced diets and deficiencies in essential nutrients. Additionally, the seasonal nature of work at tea plantations exacerbates food insecurity due to income fluctuations during lean harvest periods (9).

Recognizing these challenges, the Global Alliance for Improved Nutrition (GAIN) with support from the Ministry of Foreign Affairs of the Netherlands in collaboration with the Ethical Tea Partnership (ETP) and supported by 8 multinational tea companies initiated the “Healthy Diets for Tea Communities” intervention. This was part of a larger public-private partnership aimed at improving the nutritional quality of tea workers’ diets in three countries. The intervention sought to improve access, awareness, and consumption of healthier foods among tea workers and their families. This paper presents findings from a study designed to assess the extent to which the intervention in Assam, India was successful in changing the consumption of local vegetables and fortified oils.

The study used mixed method, repeated cross-sectional design as described in section two. Specifically, the study assessed whether there was an increase in fruit, vegetable and fortified oil consumption among the tea workers and their families, and if this contributed to measurable changes in dietary diversity. It also examined to what extent the intervention improved diet and nutrition knowledge and awareness, and whether the availability of healthier foods at the estate level improved due to supply-side interventions of the program.

## 2 Methods

### 2.1 Study design and sampling

**Figure 1:**
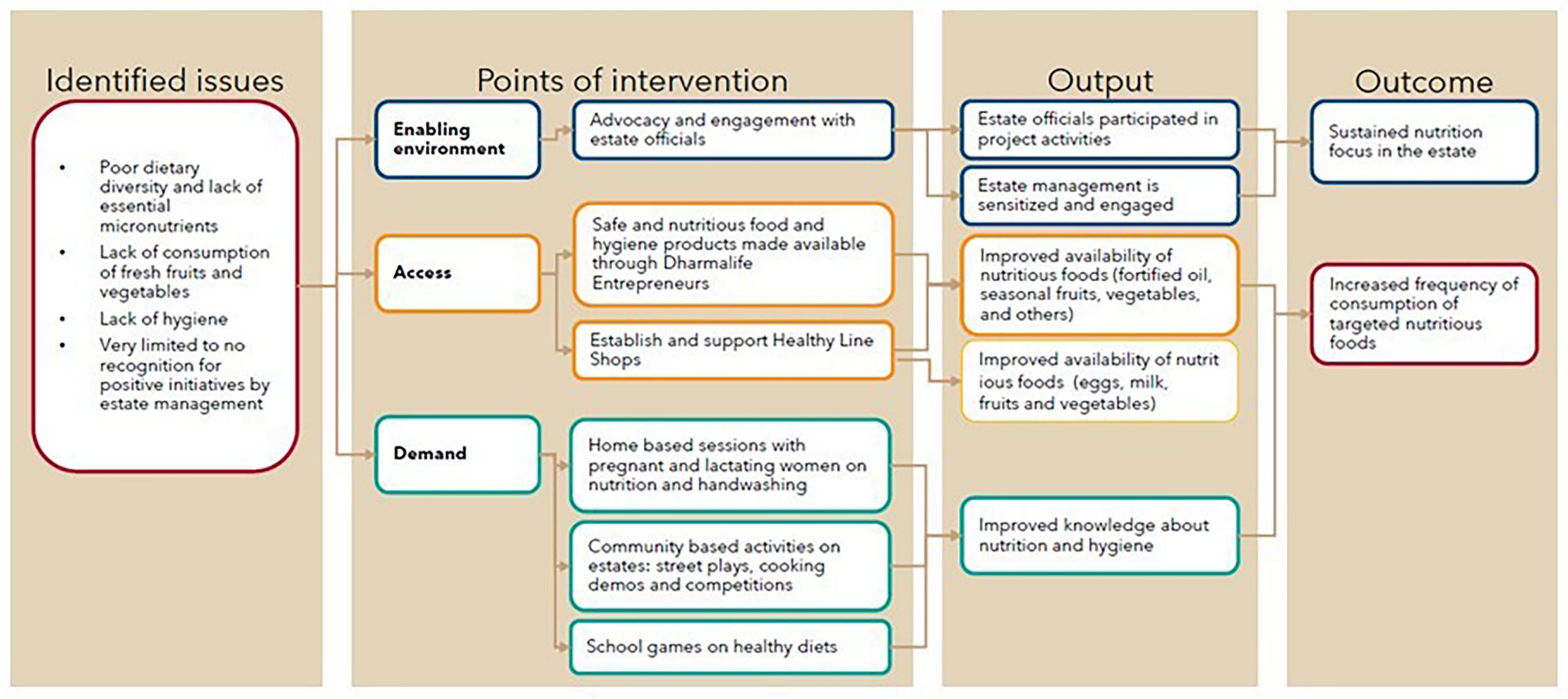
Project theory of change.

To assess the intervention’s impact, the study employed a mixed method, repeated cross-sectional design. Data on key outcomes and impact level indicators were collected from a randomly selected sample of the target population at baseline and endline—before and after the intervention- to measure changes over time. Quantitative data were collected through household surveys, while qualitative insights were gathered through key informant interviews and focus group discussions. Message recall was assessed at endline through household surveys by asking participants to identify intervention channels they were exposed to (e.g., street plays, cooking demos) and to recall key nutrition messages. Recall of messages was also explored qualitatively in FGDs. Additionally, monitoring data were analyzed within the study’s theory of change, assessing coverage, completed activities, and immediate outputs (Figure 1). For quantitative sample size estimation, a total of 989 tea workers across 66 clusters was calculated to detect an 8 percentage point increase in the proportion of women meeting minimum dietary diversity for women (MDD-W)—defined as consumption of at least five out of 10 food groups—with 95% confidence and 80% power.

The quantitative household survey used multi-stage sampling design. In the first stage, 30 tea estates were randomly selected from 36 intervention estates, representing both supply-side models. In the second stage, 2–3 divisions within each estate were randomly selected, totaling 66 divisions; and finally, in the final stage, 15 worker households were randomly selected for participation. A large tea estate is typically divided into smaller, manageable geographical units or “sections” known as division. Each division may have different tea plants, age profiles of bushes, or even microclimates, which can influence the quality and yield of the tea. These divisions are managed by a supervisor who oversees the daily plucking and cultivation activities. For qualitative data collection, the study conducted 20 key informant interviews and 10 focus group discussions. Inclusion criteria required respondents to be women aged 15–49, residing and working on the tea estate. Women were the primary study respondents as they comprise most of the tea workforce and bear a disproportionate burden of malnutrition.

### 2.2 The healthy diets for tea communities’ initiative in India

The ‘Healthy Diets for Tea Communities’ intervention aimed to enhance dietary diversity and nutrition among over 90,000 tea estate workers in Assam between 2020 and 2023. This initiative is part of GAIN’s workforce nutrition programs which targets improvements in nutrition for workers and farmers in low- and middle-income countries within agricultural and industrial supply chains, recognizing the mutual benefits of a healthier workforce to workers and businesses.

The main objective was to increase the consumption of specific food groups, notably fortified oil and local fruits and vegetables to improve overall diets. To achieve this, the intervention employed two strategies:

1. Behavior Change Communication (BCC) activities aimed to raise demand for nutritious foods among tea worker communities, focusing on promoting balanced diets, consumption of seasonal produce, and use of fortified oil for cooking. These efforts were implemented through home-based sessions, edutainment-based community events such as street plays, cooking demonstrations and competitions.
2. Supply-side interventions focused on improving access to nutritious foods to 19,000 households through one of two innovative delivery models.
3. *Door-to-door sales by Dharma life Entrepreneurs (DLEs)*: DLEs were women 21–49 years of age selected from the tea estate and trained on entrepreneurship and nutrition. They not only focused on selling fortified oil, pulses, and spices, but also sold products like solar cookers, induction cooktops, and handwash soap to ensure sufficient sales to maintain a business case for the entrepreneurs. A total of 78 Dharma life Entrepreneurs (DLEs) were trained and worked in 76 estates, to reach 6,942 households.
4. *Healthy Line Shops (HLS):* A retail shop model wherein existing kiosks were supported to offer a wider range of nutritious food products and bolstered by marketing materials. These were branded as Healthy Line Shops (HLS) in tea estates and developed in collaboration with a technical partner, Ecociate Consultants. The model helped shop owners to first source and then introduce new nutritious food products such as lentils, fortified oil, eggs, milk, vegetables, and fruits. The 152 HLSs across 112 tea estates participating had the capacity to reach 12,000 households.

### 2.3 Data collection and analysis

#### 2.3.1 Data collection

Data were collected from 33 tea estates across 4 districts in Assam: Dibrugarh, Jorhat, Tezpur, and Tinsukia. The baseline survey was conducted in February–March 2021 and the endline survey in March–April 2023 after 2 years of intervention implementation and during the same season to control for food availability. Respondents for the household interviews were female tea workers aged 15–49, responsible for household food preparation. Informed consent was obtained before each interview, and participants retained the right to terminate the interview at any time. This study was approved by the Sigma Institutional Review Board in New Delhi, India (IRB Numbers: 10051/IRB/20-21 and 10,109/IRB/22-23).

Monitoring data were also analyzed. For the DLE areas, sales performance was tracked regularly by a phone-based application. This data was part of regular communication where entrepreneurs engaged in both weekly and monthly operational calls with implementation managers to gather updates on the intervention and gain insights into sales and behavior change activities. For the HLS areas, a customized monitoring application was developed to capture sales data and behavior change activity information, aligned with the intervention’s key performance indicators (KPIs). Coordinators gathered sales data from HLS distributors by reviewing bills and records, while HLS owners were trained to maintain a stock register, documenting sales for each product and forecasting inventory needs.

#### 2.3.2 Data analysis methods

Statistical association between the two rounds of surveys was assessed using chi-square tests for categorical variables and t-tests for continuous dichotomous variables. To account for differences across survey rounds, propensity scores were calculated using logistic regression, where the independent variable was exposure to the intervention (1 = endline participants; 0 = baseline participants) and observed socio-demographic characteristics served as predictor variables. To match tea workers surveyed at baseline and endline, we used the nearest-neighbor matching with a caliper radius of 0.0015, ensuring each participant was paired 1:1 with an unexposed participant, constructing a robust counterfactual for comparison.

The overall covariate imbalance of the model was examined by testing the joint significance of all the regressors (i.e., the ability of covariates to predict exposure to any intervention) using the likelihood ratio test before and after matching. A further test of robustness and quality of the matching was conducted through a comparison of the pseudo-R-squared in pre- and post-matching (12). To estimate the difference in key outcomes between exposed and control groups we used the average treatment effect on treated (ATT). Outcomes on the overall tea worker population were weighted to reflect the proportion of Healthy Line Shops (HLS) and Dharma life Entrepreneurs (DLE) in the population reached by the program.

Key outcomes on diets (diversity and individual food group consumption) were derived from the minimum dietary diversity for women (MDD-W) indicator guidance by FAO, USAID and FANTA (13). Perceptions of food availability were measured using Likert scales. Only significant results with a value of *p* < 0.05 are presented unless stated otherwise.

## 3 Results

### 3.1 Socio-economic characteristics

A total of 989 estate workers were surveyed at the baseline and 871 at the endline. The reduced endline sample was due to entry refusals from a few tea estates under new ownership. The sociodemographic characteristics of the study population were assessed at both baseline and endline. With the exception of the number of children under five in the DLE and HLS groups, and the DLE group, all other sociodemographic characteristics showed significant differences between baseline and endline. To address these imbalances, propensity score matching (PSM) was performed, as described in the methods section, to balance the samples. Table 1 presents social demographic characteristics after matching, while Table 2 summarizes key outcome indicators. Pre-matching socio-demographic characteristics are provided as Supplementary material (Table 3).

**Table 1:** Baseline and endline socio-demographic characteristics of the participants post matching.

	DLE & HLS				DLE				HLS			
	Baseline		Endline		Baseline		Endline		Baseline		Endline	
	(*n* = 347)		(*n* = 780)		(*n* = 102)		(*n* = 356)		(*n* = 245)		(*n* = 424)	
Age (years), mean (SD)												
	32.2	8	31.2	6.6	32.9	7.6	31.7	6.1	31.9	8.2	30.8	7
*p-value^1^*	0.037**				0.103				0.079			
Marital status*, n (%)*												
Never married	4	1.2	8	1	2	2	3	0.8	2	0.8	5	1.2
Currently married	325	93.7	753	96.5	98	96.1	346	97.2	227	92.7	407	96
Widowed	16	4.6	19	2.4	1	1	7	2	15	6.1	12	2.8
Divorced/Separated	2	0.6	0	0	1	1	0	0	1	0.4		0
*p-value^2^*	0.038**				0.183				0.007**			
Respondent education*, n (%)*												
Illiterate	112	32.3	163	20.9	37	36.3	63	17.7	75	30.6	100	23.6
Literate without formal schooling	12	3.5	32	4.1	7	6.9	11	3.1	5	2	21	5
Primary	114	32.9	256	32.8	28	27.5	139	39	86	35.1	117	27.6
Middle	74	21.3	282	36.2	17	16.7	126	35.4	57	23.3	156	36.8
Higher secondary	26	7.5	43	5.5	9	8.8	17	4.8	17	6.9	26	6.1
Senior secondary	7	2	4	0.5	4	3.9		0	3	1.2	4	0.9
University/College Master	2	0.6		0		0		0	2	0.8		
*p-value^2^*	0.000**				0.000**				0.000**			
Source of household income*, n (%)*												
Agriculture	5	1.4	2	28.6			2	0.6	5	2		0
Agriculture labor	89	25.6	598	87.1			352	98.9	89	36.3	246	58
Casual labor	104	30	137	56.9	3	75	1	0.3	101	41.2	136	32.1
Salaried (Government/private)	146	42.1	42	22.3	98	99	1	0.3	48	19.6	41	9.7
Own business/kiosk	3	0.9	1	25	1	100		0	2	0.8	1	0.2
Others								0		0		0
*p-value^2^*	0.000**				0.000**				0.000**			
Household size, mean (SD)												
	4.7	1.7	5.3	1.5	4.6	1.7	5.4	1.4	4.7	1.7	5.2	1.5
*p-value^1^*	0.000**				0.000**				0.000**			
Earning member, mean (SD)												
	2.08	0.8	2.36	0.7	1.99	0.94	2.4	0.6	2.12	0.73	2.33	0.73
*p-value^1^*	0.000**				0.000**				0.000**			
Below 5 household size, mean (SD)												
	0.64	0.9	0.71	0.8	0.76	0.99	0.72	0.8	0.58	0.86	0.71	0.8
*p-value^1^*	0.148				0.65				0.059			
Wealth index, (%)												
Poor	170	49	188	24.1	52	51	53	14.9	118	48.2	135	31.8
Middle	121	35	384	49.2	34	33.3	199	55.9	87	35.5	185	43.6
Rich	56	16	208	26.7	16	15.7	104	29.2	40	16.3	104	24.5
*p-value^2^*	0.000**				0.000**				0.000**			

** Significant at 5% level; *p*-value (1) for difference between treatment and comparison groups using independent T-test; *p*-value (2) for difference between treatment and comparison using chi-square test. ND, no data available; 3 Independent samples Mann-Whitney U-test; I: intervention; C: Control; NS.

**Table 2:** Outcome indicators (Indicators for DLE & HLS).

Indicator	BASELINE	ENDLINE	ATT^a^	95% CI for ATT		*p*-Value
	(*n* = 245)	(*n* = 424)				
	%	%		Min	Max	
Minimum dietary diversity (MDD-W) for Women	49.1%	77.3%	0.28	0.22	0.34	0.0000**
Dietary diversity score	4.52	5.90	1.38	1.17	1.59	0.0000**
Perceived availability of nutritious foods score (mean)						
	10.7	10.7	−0.1	−0.2	0.1	0.3852
Grains, white roots and tubers, and plantations	99.0%	100.0%	0.0	0.0	0.0	0.0045**
Pulses (beans, peas, and lentils)	99.6%	96.9%	0.0	0.0	0.0	0.0105**
Nuts and seeds	97.0%	90.5%	−0.1	−0.1	0.0	0.0005**
Dairy products	94.8%	91.5%	0.0	−0.1	0.0	0.0799
Meat, poultry, and fish	99.7%	98.0%	0.0	0.0	0.0	0.0554
Eggs	99.6%	97.8%	0.0	0.0	0.0	0.0485**
Dark green leafy vegetables	99.7%	100.0%	0.0	0.0	0.0	0.1200
Other vitamin A-rich fruits and vegetables	99.7%	98.9%	0.0	0.0	0.0	0.2655
Other vegetables	99.8%	99.8%	0.0	0.0	0.0	0.8872
Other fruits	97.5%	99.1%	0.0	0.0	0.0	0.0429**
Fortified cooking oil	96.5%	98.5%	0.0	0.0	0.0	0.0497**
Food groups consumed in the previous day (based on 24-h dietary recall)						
1. Grains, white roots and tubers, and plantains	100.0%	100.0%	0.0	0.0	0.0	
2. Pulses (beans, peas, and lentils)	89.5%	95.3%	0.1	0.0	0.1	0.0005**
3. Nuts and seeds	14.2%	35.7%	0.2	0.2	0.3	0.0000**
4. Dairy	27.6%	41.8%	0.1	0.1	0.2	0.0000
5. Meat, poultry, and fish	65.6%	65.4%	−0.1	−0.1	0.1	0.9651
6. Eggs	23.9%	26.0%	0.0	0.0	0.1	0.4757
7. Dark green leafy vegetables	32.6%	61.2%	0.3	0.2	0.4	0.0000**
8. Other vitamin A-rich fruits and vegetables	10.5%	48.8%	0.4	0.3	0.4	0.0000**
9. Other vegetables	73.6%	97.1%	0.2	0.2	0.3	0.0000**
10. Other fruits	14.5%	18.2%	0.0	0.0	0.1	0.1550
Fortified cooking oil	56.7%	69.9%	0.1	0.1	0.2	0.0000**
Consume at least one of, Vitamin A-rich vegetables; green leafy vegetables or fortified oil	68.9%	90.4%	0.2	0.2	0.3	0.0000**

^a^Average treatment effect for the treated (ATT)/ The difference in outcomes between exposed and control groups was directly compared to show the impact of intervention in the exposed group.

**Table 3:** Baseline and endline socio-demographic characteristics of the participants pre-matching.

	DLE & HLS				DLE				HLS			
	Baseline		Endline		Baseline		Endline		Baseline		Endline	
	(*n* = 989)		(*n* = 871)		(*n* = 449)		(*n* = 435)		(*n* = 540)		(*n* = 436)	
Age (years), mean (SD)												
	32.9	7.7	31.0	6.6	33.3	7.5	31.2	6.1	32.7	7.9	30.7	7.0
*p-value^1^*	0.000				0.000				0.000			
Marital status*, n (%)*												
Never married	20	2.0	8	0.9	7	1.6	3	0.7	13	2.4	5	1.1
Currently Married	910	92.0	844	96.9	421	93.8	425	97.7	489	90.6	419	96.1
Widowed	56	5.7	19	2.2	20	4.5	7	1.6	36	6.7	12	2.8
Divorced/Separated	3	0.3			1	0.2			2	0.4		
*p-value^2^*	0.000				0.034				0.007			
Respondent education*, n (%)*												
Illiterate	386	39.0	170	19.5	195	43.4	69	15.9	191	35.4	101	23.2
Literate without formal schooling	38	3.8	37	4.2	29	6.5	15	3.4	9	1.7	22	5.0
Primary	327	33.1	282	32.4	132	29.4	163	37.5	195	36.1	119	27.3
Middle	185	18.7	328	37.7	70	15.6	166	38.2	115	21.3	162	37.2
Higher secondary	41	4.1	50	5.7	18	4.0	22	5.1	23	4.3	28	6.4
Senior secondary	9	0.9	4	0.5	5	1.1			4	0.7	4	0.9
University/College Master	3	0.3							3	0.6		
*p-value^2^*	0.000				1.470E−22				1.435E−09			
Source of household income*, n (%)*												
Agriculture	5	0.5	2	0.2			2	0.5	5	0.9		
Agriculture labor	232	23.5	684	78.5			431	99.1	232	43.0	253	58.0
Casual labor	185	18.7	139	16.0	3	0.7	1	0.2	182	33.7	138	31.7
Salaried (Government/private)	558	56.4	45	5.2	441	98.2	1	0.2	117	21.7	44	10.1
Own business/kiosk	8	0.8	1	0.1	5	1.1			3	0.6	1	0.2
Others	1	0.1							1	0.2		
*p-value^2^*	0.000				0.000				0.000			
Household size, mean (SD)												
	4.6	1.6	5.5	1.5	4.7	1.7	5.6	1.5	4.6	1.6	5.3	1.6
*p-value^1^*	0.000				0.000				0.000			
Earning member, mean (SD)												
	1.99	0.7	2.48	0.8	1.99	0.76	2.58	0.7	1.99	0.65	2.39	0.79
*p-value^1^*	0.000				0.000				0.000			
Below 5 household size, mean (SD)												
	0.69	0.96	0.73	0.8	0.79	1.06	0.74	0.8	0.61	0.86	0.72	0.8
*p-value^1^*	0.261				0.485				0.026			
Wealth index, (%)												
Poor	555	56	189	21.7	260	57.9	54	12.4	295	54.6	135	31.0
Middle	324	33	420	48.2	144	32.1	230	52.9	180	33.3	190	43.6
Rich	110	11	262	30.1	45	10.0	151	34.7	65	12.0	111	25.5
*p-value^2^*	0.000				0.000				0.000			

*p*-value (1) for difference between treatment and comparison groups using independent T-test; *p*-value (2) for difference between treatment and comparison using chi-square test. ND, no data available; 3 Independent samples Mann-Whitney U-test; I: intervention; C: Control; NS.

### 3.2 Awareness of healthy diets and nutritious foods

Baseline data indicated a high level of awareness on diets with 90% of respondents already knowing about the benefits of seasonal fruits and vegetables, and this did not change significantly by the end of the intervention. Qualitative focus groups revealed that tea workers associate fresh produce with essential nutrients like calcium, iron, vitamins, minerals, and protein, and understood the role of nutritious foods in maintaining health and energy levels.

The proportion of respondents who had heard of fortified oil increased significantly from 68.2% at baseline to 82.9% at endline (*p* < 0.05). The study also looked at the sources of information on healthy eating habits within the target communities. Familial connections, such as family members (49.9%) and neighbors/friends (71.3%), remained the most consistent source of information at both baseline and endline. However, the interventions including cooking competitions (32.3%), demonstrations (56.3%), and home visits (54.8%) emerged as new information sources by the endline survey.

The evaluation also revealed a positive association between intervention exposure and improved message recall, particularly among women. To assess exposure and message recall, women were asked to recall specific nutrition messages after participating in behavior change activities. Among those who attended cooking demonstrations or competitions, recall of key messages was high: 87% recalled messages about using fortified oil, 98% recalled recommended food preparation methods, and 95% recalled messages on the importance of seasonal vegetables.

### 3.3 Access to nutritious foods

**Figure 2:**
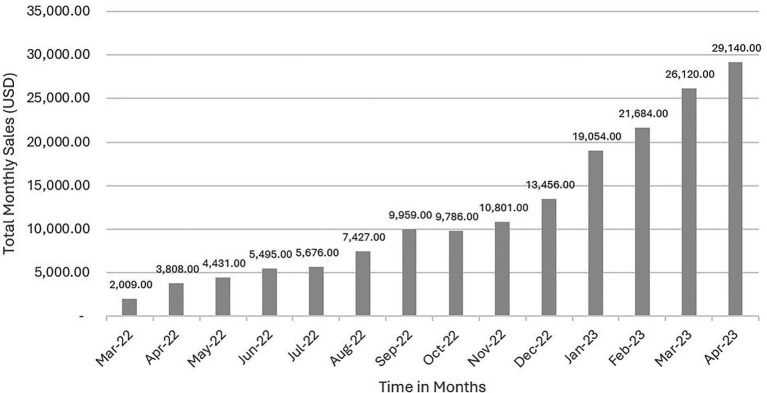
Monthly sales of nutritious food items across all Healthy Line Shops (HLS), March 2022 to April 2023. Values represent total sales for each individual month, across 152 HLS outlets participating in the intervention.

This study assessed changes in access to the targeted nutritious foods through both the household survey as well as monitoring data on sales in both the Dharma life Entrepreneurs (DLE) and Healthy Line Shops (HLS) models. Based on available monitoring data, 120 Healthy Line Shops (HLS) across four districts in Assam participated in the intervention. The aggregated sales value of nutritious food products grew from INR 166,759 (USD 2,009; 1 USD = 83.00 INR on 31 March 2022) to INR 2,427,457 (USD 29,246; 1 USD = 83.00 INR on 30 April 2023), refer to Figure 2. On average, the 120 HLSs sold INR 316,074 (USD 3,808) worth of products per month. Most of the sales (60%) were from intervention-promoted nutritious food products such as fortified mustard oil, fortified refined oil, milk, eggs, pulses (lentils, white peas), and iodized salt.

**Figure 3:**
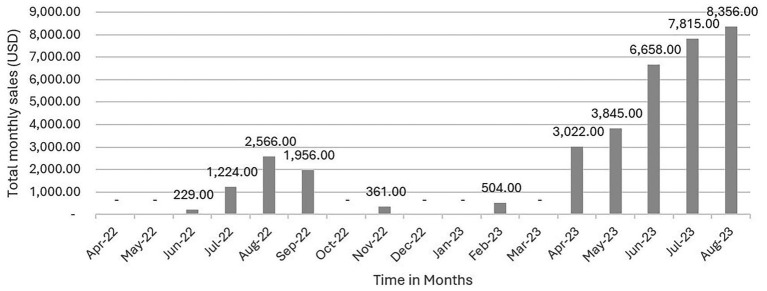
Monthly sales of nutritious food items by Dharma Life Entrepreneurs (DLEs), June 2022 to August 2023. Values represent total sales for each individual month, aggregated across all 78 DLEs participating in the intervention.

Program monitoring data on sales made by DLEs were also reviewed. In total, 78 DLEs were trained and deployed in 76 estates. Data from April 2023 indicate total sales of INR 250,787 (USD 3,021) which doubled by August 2023 to INR 693,531 (USD 8,356) (Figure 3). The supply-side approach involving door-to-door sales by DLEs and sales at the tea estate level by HLS aimed at addressing the availability aspects of the intervention. When asked about the 10 food groups and their availability, all food groups were ‘always available’ or “sometimes available” to at least 85% of the respondents. When asked about fortified oil, among households served by DLEs fortified oil was said to be available by 67% of respondents at baseline and 97% by the endline. Among households served by HLS’ there was high availability at baseline (97%) and endline (99%).

### 3.4 Dietary outcomes

#### 3.4.1 Consumption of targeted food groups

The survey assessed the changes in consumption of fortified oil and vegetables over the period of the intervention. At the household level, there was a 13.2% increase in the use of fortified oil. By intervention type, a 60.5% increase was observed in the DLE and a 13.5% increase in the use of fortified oil was observed in the HLS (Table 4). The proportion of women consuming dark green leafy vegetables (DGLVs) increased by 28.6%. When disaggregated by intervention model, a 34.5% increase was observed in the DLE and a 29.0% increase of the consumption of DGLVs was observed in the HLS (Table 4).

**Table 4:** Dietary diversity scores, minimum dietary diversity for women, and consumption of targeted foods, overall and by model (HLS and DLE).

	Indicator			MDD-W (%)	DDS	Fortified cooking oil (%)	Dark green leafy vegetables (%)	Other vitamin A-rich fruits and vegetables (%)
Overall	BL	*N* = 245		49.1	4.52	57.0	32.6	11.0
	EL	*N* = 424		77.3	5.9	70.0	61.2	49.0
	ATT			28.2	1.38	13.2	28.6	38.3
	95% CI for ATT		Min	22.0	1.17	7	22.0	32.0
			Max	34.0	1.59	20	35.0	45.0
	*p*-Value			0.000**	0.000**	0.00	0.00	00
HLS	BL	*N* = 245	%	43.0	4.29	53.1	27.6	16.0
	EL	*N* = 424	%	81.0	5.98	66.6	56.6	46.2
	ATT			38.0	1.69	13.5	29.0	30.0
	95% CI for ATT		Min	31.0	1.45	5.9	21.0	23.0
			Max	45.0	1.92	21.1	37.0	37.0
	*p*-Value			0	0	0.00	0.00	0.00
DLE	BL	*N* = 102	%	26.3	4.3	11.3	31.9	5.1
	EL	*N* = 356	%	73.1	5.8	71.8	66.4	50.6
	ATT			50.0	1.5	60.5	34.0	46.0
	95% CI for ATT		Min	40.0	1.2	51.1	24.0	36.0
			Max	60.0	1.9	69.9	45.0	56.0
	*p*-Value			0	0	0.00	0.00	0.00

** Significant at 5% level; MDD-W; Minimum Dietary Diversity; DDS-Dietary Diversity Score: BL; BL, EL; Endline, ATT (average treatment effect on the treated).

Similarly, the intake of vitamin A-rich fruits and vegetables increased by 38.4%. By intervention type, an increase of 45.6% was observed in the DLE model, and an increase of 30.2% in the HLS model (Table 4). Overall, there was a 21.5% increase (from 68.9 to 90.4%) in the proportion of respondents who consumed at least one of the targeted foods (vitamin A-rich fruit or vegetable; green leafy vegetables or fortified oil) (Table 2).

#### 3.4.2 Dietary diversity and minimum dietary diversity for women

**Figure 4:**
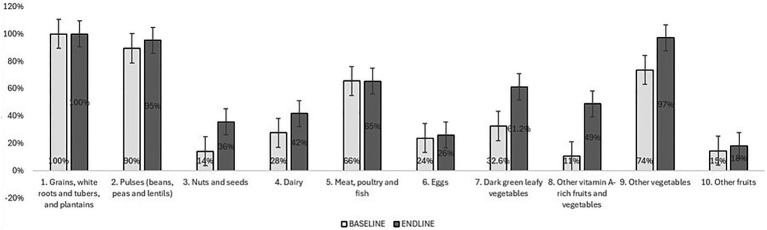
Proportion of food consumed yesterday, by food group (Post Matching).

**Figure 5:**
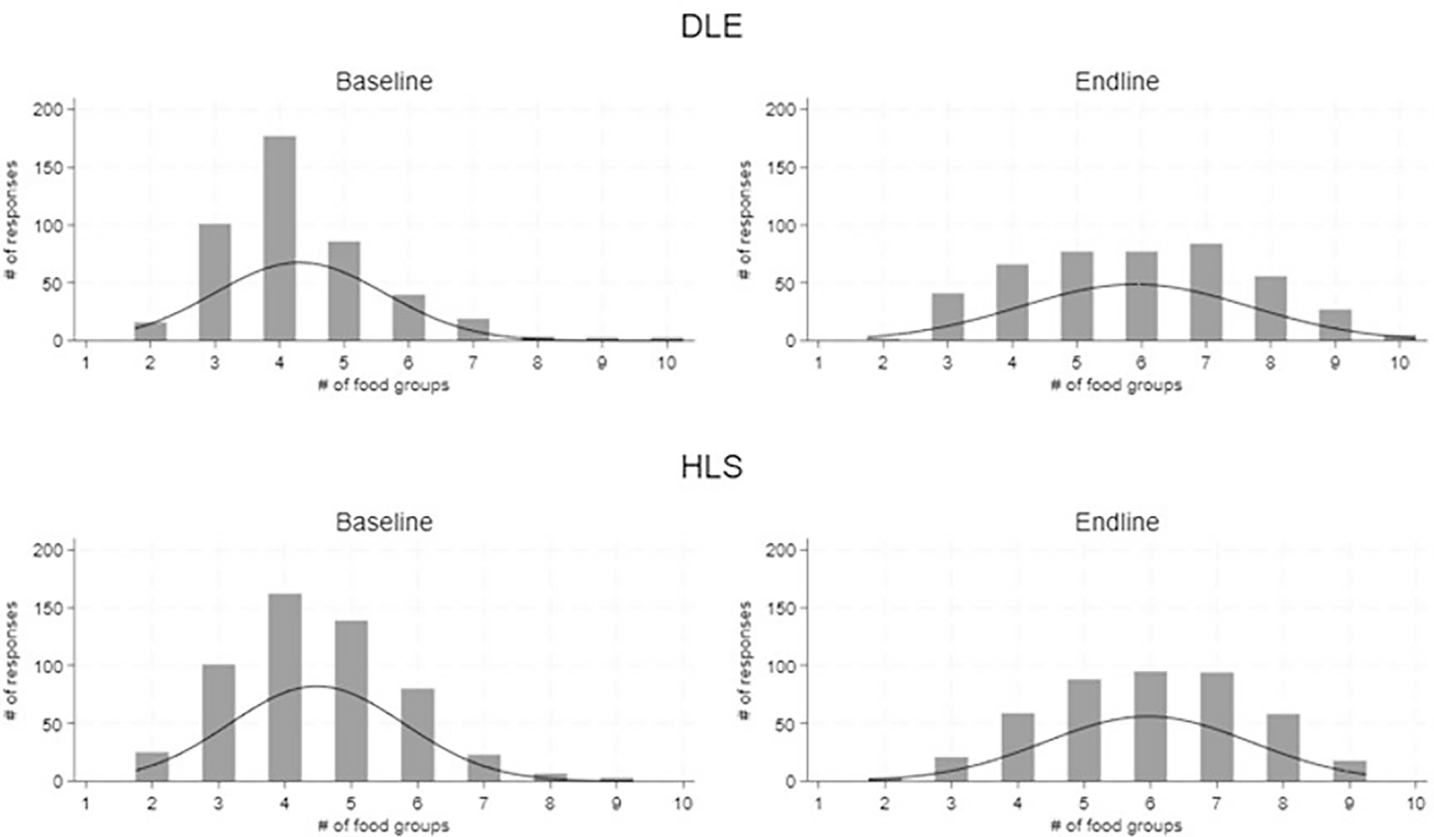
Distribution of women aged 15–49 consuming 1–10 food groups in the previous 24 h, at baseline and endline, disaggregated by supply model [Healthy Line Shops (second row) and Dharma Life Entrepreneurs (first row)]. Data are presented post matching.

The study assessed whether the promotion of targeted foods had a measurable impact on dietary diversity. Changes in the average dietary diversity score (DDS) and minimum dietary diversity for women (MDD-W) are shown in Table 4. Overall and across all models, the DDS and MDD-W increased significantly (*p* < 0.01). Specifically, the average DDS increased from 4.52 to 5.90 (+1.38) [foods groups]. This was similar across the models, DDS increased by +1.5 with DLEs and by +1.69 with the HLSs. Figure 4 illustrates a general increase in the number of food groups consumed the previous day (out of 10) from baseline to endline. The proportion of women who met minimum dietary diversity increased by 38.0% among the Healthy Line Shops target group and 50.0% among the Dharma life Entrepreneurs target group. Overall, in both groups, the proportion increased by 28.2 percent. The improvements in DDS and MDD-W scores were driven by increases in consumption of 4 food groups, in particular: dark green leafy vegetables (DGLVs), vitamin A-rich vegetables and fruits, dairy, and nuts (Figure 5).

## 4 Discussion

The interventions were implemented during the COVID-19 pandemic and were subject to the related disruptions. Recent literature that was published during or after the COVID-19 pandemic highlights how the pandemic has impacted markets, food security, dietary diversity, and food purchasing behaviors, both at country level [for example, in Pakistan (14)] as well as through review studies (15). The systematic review by Picchioni et al. (15) raises concerns about how access to and affordability of healthy diets is impacted for women and individuals with a low socio-economic status by economic and health crisis (such as those triggered by the COVID-19 pandemic). This study contributes to existing literature by evaluating the effectiveness of two market-based approaches to improving diet quality among tea workers, with a focus on systemic and sustainable improvements. Unlike studies that primarily examine food security and diets in the context of acute shocks such as COVID-19, or look into more isolated approaches such as micronutrient supplementation (16) this research explored longer-term market-based strategies to strengthen food environments and diet quality in a structurally vulnerable population group.

The evaluation results indicate that the interventions on tea estates were associated with a significant increase in the proportion of women meeting minimum dietary diversity (MDD-W)—rising from approximately 50% at baseline to over 75% at endline. This improvement was driven by increased consumption of dark green leafy vegetables, vitamin A-rich fruits and vegetables, pulses, nuts and seeds, other vegetables, and fortified oils.

### 4.1 Awareness and demand creation

In terms of awareness of healthy diets and nutritious foods, the survey found that most respondents (90%) were aware of the benefits of seasonal fruits and vegetables at baseline, and it did not change significantly over the intervention period. There was, however, increased awareness of fortified oil. As suggested by the literature, knowledge alone is not sufficient for changing dietary behavior (17). We believe that, in our context, barriers such as low motivation, limited willingness, and access constraints played a larger role in hindering dietary improvements as opposed to a lack of knowledge. As such, the SBCC interventions were designed to go beyond raising awareness by actively encouraging behavior change through interactive methods like community dramas, cooking demos, and food-related contests.

A previous program by GAIN in Assam tea estates had limited effect on improving dietary diversity in the population (18). The study concluded that a SBCC only approach was a shortcoming in the design, which prompted the implementation of supply side models in this program (18). The previously studied SBCC approach lasted 9 weeks and focused on small group sessions for women, whereas the current intervention provided year-long community-wide edutainment, including street plays and cooking demonstrations. High recall of messages from interactive intervention activities suggests a successful behavior change approach which may have been more motivational than the previous intensive small group sessions. The heightened sales of nutritious items, including fruits, vegetables, pulses, and dairy were likely propelled by these intervention-related demand activities. However, it is also likely that some dietary shifts were due to changes in affordability of foods. The baseline was conducted in March 2021, right after COVID-19 lockdowns (which affected food affordability and availability) were lifted in the state of Assam.

### 4.2 Healthy Line Shops (HLS) and Dharma Life Entrepreneurs (DLE): supply-side interventions

The intervention implemented two market-based supply-side approaches: the HLS or DLE model in each tea estate. Both models aimed to improve access to nutritious foods through innovative delivery models and did not rely on free or subsidized provisions. While the tea workers were of low socioeconomic status they still can and do buy their food.

The HLS model transformed existing kiosk shops into outlets offering a range of nutritious food products serving as centralized hubs within the estates. The DLE model, in contrast, introduced a door-to-door sales mechanism operated by trained women entrepreneurs, offering personalized outreach and delivery of nutritious foods and household products.

Both models achieved measurable improvements in access and sales. Sales of nutritious foods increased eight-fold under the HLS model and three-fold under the DLE model. The DLE model, being a novel sales mechanism in this region, also generated new livelihood for 78 women in the communities. While both approaches disseminated nutrition and health messages, their modes of delivery varied—HLSs functioned as fixed-location access points for nutritious food, whereas DLEs enabled customized and interpersonal interactions with households.

From a comparative standpoint, the DLE model appeared more effective in increasing the consumption of targeted foods and demonstrated stronger gains in dietary diversity indicators. Its flexible, relationship-based approach allowed better responsiveness to community preferences and needs, though it came at a higher cost per beneficiary. The HLS model, on the other hand, required greater initial investment but offered broader geographic reach and stronger potential for integration into existing supply chains, benefitting not only consumers but also local traders and wholesalers. While HLSs may offer advantages in scale and sustainability, DLEs proved valuable in areas where trust, convenience, and behavior change were critical to adoption.

Notably, by the end of the program period, both models were operating without external financial support—providing indication of their potential sustainability. Their continuation, however, is dependent on sustained demand from consumers.

Besides the HLS and DLE models, there is room for other market approaches to improve access to nutritious foods and healthier diets. For instance, Unilever’s Shakti Amma intervention (19) empowers women as micro-entrepreneurs selling a range of products, including some that promote better health and nutrition, has also demonstrated sales improvements. Such models highlight the broader potential for reaching low-income consumers—who collectively represent a vast market—with nutritious products in a financially viable manner (20).

The study findings highlight the critical role that market-based innovations and approaches can play in improving diets among tea estate workers. This underscores the need for integrating workforce nutrition into health and labor policies especially in the agricultural sectors such as tea estates that employ large numbers of women. These findings suggest that policymakers should embed behavior informed strategies into nutrition policies targeting labor-intensive sectors. Managers in tea estates should prioritize sustainable partnerships with local vendors and nutrition stakeholders to promote sustainable access to healthy diets at the workplace. Also integrating motivational messaging with deliberate improvements in food availability can reinforce behavior change hence supporting long-term dietary improvements and worker productivity.

### 4.3 Limitations of the study

The analysis identified statistically significant variations in income sources and educational attainment between the baseline and endline samples. These temporal changes may have influenced the ability of intervention participants to access essential resources, nutritious foods, healthcare services, and educational opportunities. Consequently, these shifts could have had indirect effects on overall health outcomes of the population (21). The observed increase in income sources and educational levels may reflect a gradual recovery from the disruptions of COVID 19, which were particularly pronounced at the start of the intervention, as evidenced by shutdowns in two tea estates. While propensity score matching (PSM) was employed to control demographic differences when assessing the intervention’s impact, it does not establish causal relationships in the absence of a case–control study.

The prevalence of anemia is high among the target group of this study, as outlined in the introduction. However, the research questions and outcome measures of this study centered on changes in dietary diversity and overall diet quality, with MDD-W as a key outcome indicator. Anemia outcomes were not assessed in this study. While improved dietary diversity is linked to enhanced overall nutrient adequacy, it does not necessarily ensure increased iron intake or improvements in iron status, and we cannot draw conclusions about the intervention’s impact in that regard.

## 5 Conclusion

This study establishes that multi-pronged, integrated intervention- combining demand generation activities, strategic advocacy and supply supply-side market innovations can significantly improve diet quality among female workers and their households in Assam’s tea estates. The intervention was designed to address both consumer behavior and market dynamics, recognizing that nutrition outcomes are shaped by a complex interplay of availability, affordability and awareness. One of the most notable findings was observed in the improvement in dietary diversity, particularly the consumption of key nutritious foods such as vegetables. These improvements were closely linked to the increased availability and promotion of key nutritious foods, particularly vegetables, through strengthened local vendor networks. These vendors were supported through training on business outcomes and direct connections to the supply chains (doorstep delivery of nutritious foods), enabling them to stock and regularly offer a wider variety of affordable, healthy food. By strengthening these local food access points, the intervention helped build a more reliable and inclusive food environment for estate communities. On the demand side, a variety of community—based ‘edutainment’ activities—including cooking demonstrations, street plays, home-based sessions and school games on healthy diets proved to be effective in increasing motivation and willingness to purchase and consume these foods. These activities not only conveyed the essential knowledge about a balanced diet but also created opportunities for participants to engage directly in food preparation and decision making in a culturally relevant and engaging manner. This practical, participatory approach appeared to enhance community interest and confidence in adopting improved dietary habits.

Together, these efforts suggest that behavior change is more likely to be successful when awareness building is coupled with practical exposure and supportive market structures. The intervention’s ability to engage both consumers and vendors, including the healthy line shops—appears to have influenced purchasing patterns in a meaningful way, fostering an enabling food environment in a resource-constrained, labor-intensive setting. The intervention successfully established a model that continued to operate even after the project’s exit, with local vendors maintaining the supply and promotion of nutritious foods independently. This demonstrates that, once strengthened, local markets can sustain the availability, accessibility, and affordability of nutritious foods, supporting lasting improvements in dietary behavior. However, although the model promises much for long-term reproducibility and impact, there are some areas of weakness. Foremost among these is that more evidence is required to determine how far such shops adapt to shifts in consumer demand, price instability, or supply chain disruption in the long term. In addition, it is uncertain whether behavior change will accumulate or plateau in the long run without continued reinforcement. Future assessments must examine these aspects—such as local supply chain resilience, vendor profitability, and the consistency of healthy food choice among consumers—to ascertain not only that the model survives but also that it continues to evolve to meet long-term community needs.

## Data Availability

The original contributions presented in the study are included in the article/supplementary material, further inquiries can be directed to the corresponding author.
